# Procalcitonin as a Candidate Biomarker for Malarial Infection and Severe Malaria: A Meta-Analysis

**DOI:** 10.3390/ijerph191811389

**Published:** 2022-09-09

**Authors:** Aongart Mahittikorn, Kwuntida Uthaisar Kotepui, Wanida Mala, Polrat Wilairatana, Manas Kotepui

**Affiliations:** 1 Department of Protozoology, Faculty of Tropical Medicine, Mahidol University, Bangkok 10400, Thailand; 2 Medical Technology, School of Allied Health Sciences, Walailak University, Tha Sala, Nakhon Si Thammarat 80160, Thailand; 3 Department of Clinical Tropical Medicine, Faculty of Tropical Medicine, Mahidol University, Bangkok 10400, Thailand

**Keywords:** malaria, *Plasmodium*, procalcitonin, severe malaria

## Abstract

Procalcitonin (PCT), as a marker of malaria severity, remains to be investigated. The present study collated and compared the levels of PCT between patients with severe malaria, uncomplicated malaria, and control participants to assess their role in predicting malaria infection and disease severity. The systematic review was registered at PROSPERO with registration number CRD42021297243. The search for relevant studies that reported PCT in patients with malaria was performed in PubMed, Scopus, and Web of Science. The following meta-analyses were conducted; (1) the pooled mean PCT levels in patients with severe and uncomplicated malaria, and (2) the pooled mean difference in PCT levels between patients with severe and uncomplicated malaria. Fifteen studies were included for qualitative and quantitative syntheses. The meta-analysis results show that the pooled mean PCT levels in patients with uncomplicated malaria were 3.92 ng/mL (95% CI: 2.26–5.58 ng/mL, I^2^: 96.5, five studies), whereas the pooled mean PCT levels in patients with severe malaria were 14.13 ng/mL (95% CI: 8.75–19.5 ng/mL, I^2^: 92.6, six studies). The meta-analysis showed that patients with severe malaria had an equal mean of PCT compared to those with uncomplicated malaria when the random-effects model was used (*p*: 0.055, weighted mean difference: 6.93, 95% CI: −0.16–14.02, I^2^: 84.6%, four studies). There were probable correlations between the level of parasitemia, immunity level, and possibly bacterial or other parasitic co-infection that could affect the PCT level among different clinical severities of malaria. Therefore, the PCT level alone does not seem to be a suitable biomarker to discriminate the severe/uncomplicated or infected/uninfected cases. Further studies should investigate the increased PCT levels in combination with other markers in association with malaria infection and severity.

## 1. Background

Malaria is a protozoan disease caused by the *Plasmodium* species including *P. falciparum*, *P. vivax*, *P. malariae*, *P. ovale curtisi*, *P. ovale wallikeri*, and *P. knowlesi* [1]. The World Health Organization (WHO) revealed that 241 million malaria cases and 627,000 million malaria deaths were estimated in 2020 which were increased from 2019 [2]. Although *P. falciparum* is the main cause of severe malaria, *P. knowlesi* [3], *P. vivax* [4], *P. malariae* [5], and *P. ovale* [6] can also cause severe malaria but in fewer cases. The misidentification of *P. ovale* as *P. vivax* or *P. knowlesi* as *P. malariae* is frequent and leads to underreported *P. ovale* and *P. knowlesi* cases [7,8]. In addition, other tropical diseases are reported in patients with malaria as a co-infection, making the clinical diagnosis difficult [9,10,11,12,13,14]. Moreover, the decision for the treatment of malaria relies on clinical assessment. Therefore, it might result in inaccurate identification of patients with severe malaria who need more high-level monitoring, such as parenteral therapy, in the case of clinicians who have little experience or are less skilled in making the decision. Rapid diagnostic tests (RDTs) are currently playing a role in identifying patients with malaria as “point-of-care” testing; however, a point-of-care test for patients with severe malaria that does not require technical equipment or support is still being investigated.

Alterations of inflammatory markers have been investigated to predict malaria infection and follow-up on malaria severity, such as using plasma lactate [15], copeptin [16], neopterin [17], and c-reactive protein (CRP) [18,19,20] as these molecules are simple laboratory-based parameters. PCT is an amino acid precursor of the hormone calcitonin and is secreted by thyroid C cells [21]. In healthy individuals, the PCT level is relatively low (<0.1 ng/mL) [22]. Previously, PCT was described as a marker of sepsis as its level increased in response to severe systemic inflammation, particularly bacterial infections [23]. The most recent systematic review showed that increased CRP levels were a biomarker for malaria infection and monitoring of malaria severity [19,20]. However, the role of PCT as a marker of malaria infection or severe malaria is still not clear. As the knowledge of PCT levels in different clinical severities of malaria can be used in malaria diagnosis and management, the present systematic review aimed to collate the evidence of PCT in malaria and compare the levels of PCT between patients with severe malaria, uncomplicated malaria, and control participants to assess their role in predicting the malaria infection and disease severity.

## 2. Methods

### 2.1. Protocol and Registration

This systematic review was written under the preferred reporting items for systematic reviews and meta-analyses (PRISMA) for systematic review protocols [24]. This systematic review was registered at the International Prospective Register of Systematic Reviews (PROSPERO; registration number: CRD42021297243).

### 2.2. Eligibility Criteria

Qualitative and quantitative studies that reported PCT in patients with malaria were included. PICO was applied to the eligibility criteria as P: participants were patients with malaria; I: outcome of interest was PCT; C: controls were patients with uncomplicated malaria or control participants; O: the outcome was the difference in mean PCT between groups of participants. Studies were selected according to the inclusion and exclusion criteria. The inclusion criteria were: (1) studies published in the English language; (2) study designs could be clinical trials, longitudinal studies, case-control studies, cohort studies, cross-sectional studies, or observational studies; and (3) only human studies of patients with malaria infection by laboratory diagnoses such as microscopic diagnosis, molecular diagnosis, or rapid diagnostic tests. The exclusion criteria were the following: (1) reported PCT in non-malaria diseases, (2) review articles, (3) case reports/case series, (4) incomplete data/unable to extract, (5) full-texts unavailable, (6) PCT in patients co-infected with malaria and other diseases, (7) studies using the same group of participants, and (8) non-English-language studies.

### 2.3. Information Sources and Searches

The searches were performed in PubMed, Scopus, and Web of Science without restriction by publication date. Search terms included the text words malaria (OR *Plasmodium*) and procalcitonin (or PCT) (Table S1). In addition, the reference lists of included studies, review articles, and Google Scholar were also searched for gray literature to ensure that the relevant studies were not missed during the searches or study selections. The searches began on 1 December 2021 and finished on 8 December 2021.

### 2.4. Study Screening and Selection

For study screening, the titles and abstracts of the studies retrieved from the databases were screened, and then non-relevant studies were excluded. Next, the full texts of the relevant studies were examined according to eligibility criteria, and studies that did not meet the criteria were excluded for specific reasons. Then, studies were included if they met the whole eligibility criteria. Two of the authors (AM and MK) independently selected the studies for inclusion. Another author served as a third rater to create a consensus on any discrepancies in study selection between the two review authors.

### 2.5. Data Extraction

The two reviewers (MK and KUK) independently extracted data from the included studies to an Excel spreadsheet. The following data were extracted: authors, publication year, country, study design, characteristics and number of participants, *Plasmodium* spp., mean age, age groups, male percentage, PCT levels (ng/mL), parasite density, methods for malaria detection, and methods for PCT measurement. The data were then cross-checked by other authors in order to guarantee accuracy. 

### 2.6. Assessment of Risk of Bias

To evaluate the methodological quality of the included studies, the Joanna Briggs Institute (JBI) critical appraisal tools for observational studies were used [25]. Studies were assessed for risk of bias in terms of the following characteristics: inclusion criteria, study subjects and the setting, measurement of exposure, confounding factors, measurement of outcomes, and appropriate statistical analysis. Each study was rated according to the overall quality of evidence as high (7–8 scores), moderate (4–6 scores), or low (<4 scores). The discrepancies in rating between authors were resolved through consensus.

### 2.7. Data Synthesis and Statistical Analysis

The data from all included studies were narratively synthesized to provide narrative information on the characteristics of the included studies and knowledge of PCT in semi-quantitative data. For quantitative analysis, forest plots were generated to summarize the data of PCT from the included studies. The following meta-analyses were conducted: (1) the pooled mean PCT levels in patients with uncomplicated malaria; (2) the pooled mean PCT levels in patients with severe malaria; and (3) the pooled mean difference in PCT levels between patients with severe and uncomplicated malaria. The meta-analyses were conducted using the Stata software version 14.0 (College Station, TX, USA: Stata Corp LP). The heterogeneity among the included studies was considered, and a random-effects meta-analysis was used to pool the evidence. For the meta-analysis of the difference in PCT levels between two patients, the weighted mean differences (WMD) were effect estimates (ES). Cochran’s Q and I^2^ statistics were used for determining whether the heterogeneity of the included studies signified a high variation across the exhibited studies. In the absence of statistically significant heterogeneity, the effect estimates were pooled using the random-effects model [26]. The robustness of the meta-analysis results was analyzed by conducting sensitivity analyses using a fixed-effects model and the leave-one-out cross-validation technique [27].

## 3. Results

### 3.1. Search Results

**Figure 1:**
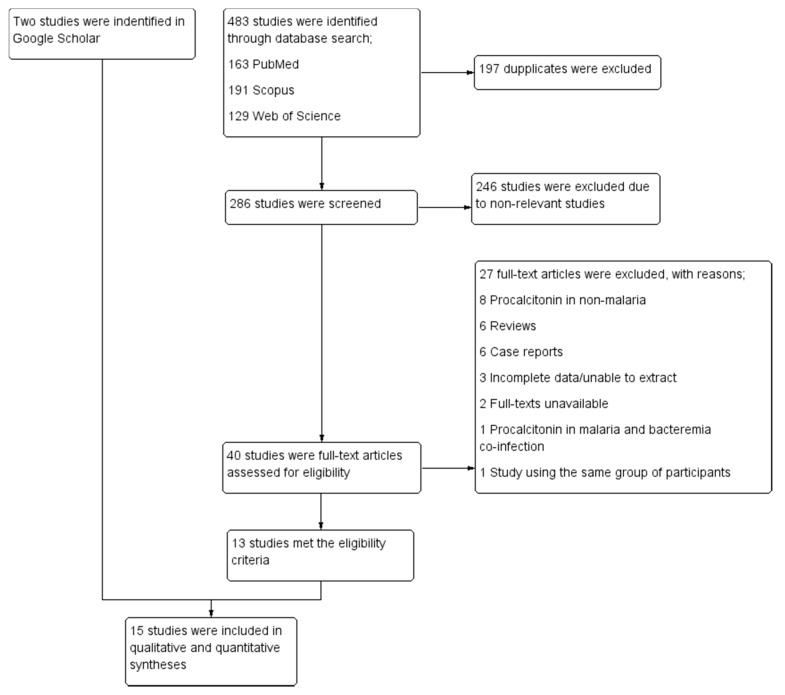
Study flow diagram demonstrating the selection of relevant studies.

In total, 483 studies were identified through 3 databases, including 163 studies from PubMed, 191 studies from Scopus, and 129 studies from the Web of Science. After 197 duplicates were removed, 286 studies remained for the title and abstract screening. The screening of the remaining studies led to the exclusion of 246 non-relevant studies. Then 40 potentially relevant studies were examined for full-texts, and 27 full-text articles were excluded for the following reasons: 8 were PCT levels in non-malaria, 6 were reviews, 6 were case reports, 3 had incomplete data/unable to extract, 2 had unavailable full-texts, 1 studied PCT in malaria and bacteremia co-infection, and 1 study used the same group of participants. Thirteen studies [16,28,29,30,31,32,33,34,35,36,37,38,39] met the eligibility criteria and were included. Another search in Google Scholar found two studies [40,41], that met the eligibility criteria and were also included. Finally, 15 studies [16,28,29,30,31,32,33,34,35,36,37,38,39,40,41] were included in the systematic review for qualitative and quantitative syntheses (Figure 1).

### 3.2. Characteristics of the Included Studies

The included studies were published between 1998 and 2019 (Table 1). Studies conducted in Africa included studies from Ghana [28], Uganda [37], Senegal [40]; and those from Asia included studies from India [41], China [31], Thailand [36], Cambodia/Laos/Thailand [38], and Indonesia [32]. Studies with multiple countries included Congo, Angola, Pakistan, and African countries [34], and studies focusing on imported malaria in Europe included France [30,35], Netherlands [16,39], Italy [29], and Germany [33]. Study designs were case-control studies (3/15, 20%) [28,36,37], cohort studies (3/15, 20%) [16,30,32], prospective observational studies (6/15, 40%) [29,33,35,39,40,41], and retrospective studies (3/15, 20%) [31,34,38]. Ten studies (10/15, 66.7%) [16,28,29,30,33,34,35,37,39,41] evaluated PCT levels in patients with severe malaria and uncomplicated malaria. Three studies [31,36,40] and two studies [32,38] evaluated PCT levels in patients with severe malaria only, and uncomplicated malaria only, respectively. Most of the included studies (10/15, 66.7%) [28,29,30,31,33,35,36,37,40,41] enrolled patients with *P. falciparum* malaria, whereas three studies [16,34,39] enrolled patients with *P. falciparum* and non-*P. falciparum* (*P. vivax*, *P. ovale*, *P. malariae*), and two studies [32,38] did not specify the *Plasmodium* spp. Most of the included studies enrolled adults (8/15, 53.3%) [16,29,30,34,35,36,39,41], and a few studies enrolled children [28,37]. Some studies did not report the age range of the participants [31,32,33,38,40]. 

### 3.3. Risk of Bias among the Included Studies

Most of the included had a low risk of bias (13/15, 86.7%) [16,28,29,30,31,33,34,35,36,37,39,40,41], whereas only two studies [32,38] had a moderate risk of bias (Table S2). There was no study with a high risk of bias; therefore, all studies were included for syntheses.

### 3.4. Mean PCT Levels in Patients with Uncomplicated and Severe Malaria

The pooled mean PCT levels in patients with uncomplicated malaria were estimated using five studies [16,28,29,30,33]. The results of the meta-analysis showed that the pooled mean PCT levels in patients with uncomplicated malaria were 3.92 ng/mL (95% CI: 2.26–5.58 ng/mL, I^2^: 96.5, five studies, Supplementary Figure S1). 

**Figure 2:**
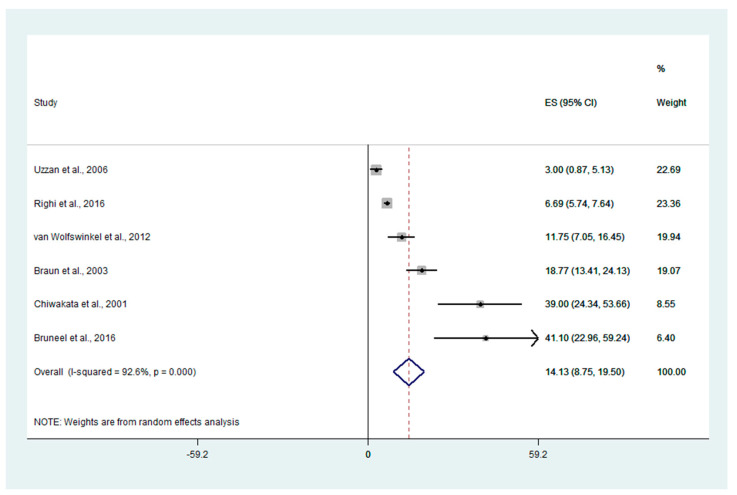
Forrest plot demonstrating the pooled mean PCT levels (ng/mL) in patients with severe malaria. Abbreviation: ES, effect estimate (pooled mean PCT); CI, confidence interval. Explanation of the forest plot: black diamond symbol: point estimate; dashed line: pooled mean PCT levels; I^2^, level of heterogeneity; *p* = 0.00 or less than 0.05, significant heterogeneity [16,28,29,30,33,35].

The pooled mean PCT levels in patients with severe malaria were estimated using six studies [16,28,29,30,33,35]. The meta-analysis results showed that the pooled mean PCT levels in patients with severe malaria were 14.13 ng/mL (95% CI: 8.75–19.5 ng/mL, I^2^: 92.6, six studies, Figure 2).

### 3.5. Differences in PCT Levels between Severe and Uncomplicated Malaria

For qualitative synthesis, the included studies revealed that PCT levels were frequently over normal limits in patients with malaria [32,34,35]. In addition, PCT levels were frequently over normal limits in patients with severe malaria [31,36,39,41]. Further, the PCT levels were frequently over normal limits compared to uncomplicated malaria [37,39,41].

**Figure 3:**
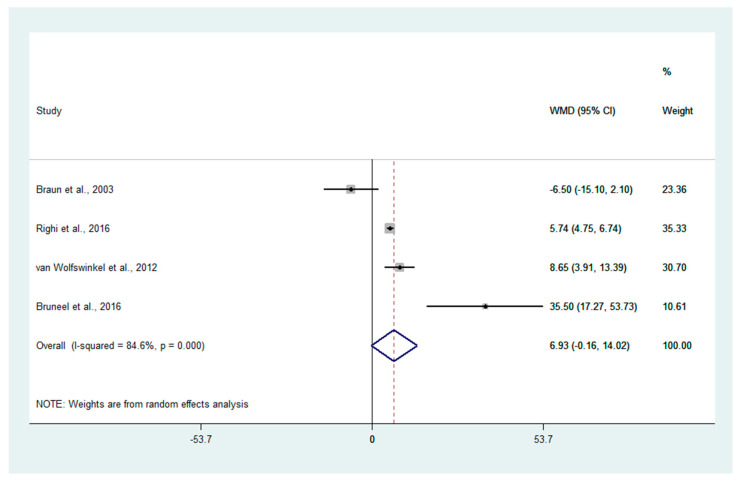
Forrest plot demonstrating the difference in the mean PCT levels (ng/mL) between patients with severe malaria and uncomplicated malaria. Abbreviation: WMD, weighted mean difference; CI, confidence interval. Explanation of the forest plot: black diamond symbol: point estimate; dashed line: WMD of PCT levels; I^2^, level of heterogeneity; *p* = 0.00 or less than 0.05, significant heterogeneity [16,28,29,30].

For quantitative synthesis, the higher mean of PCT in patients with severe malaria than those with uncomplicated malaria was demonstrated in three studies [16,29,30]. Meanwhile, one study [28] demonstrated no difference in the mean PCT levels between the two groups. The meta-analysis of four studies [16,28,29,30] by the random-effects model showed that patients with severe malaria had an equal mean of PCT compared to those with uncomplicated malaria (*p*: 0.055, WMD: 6.93, 95% CI: −0.16–14.02, I^2^: 84.6%, four studies, Figure 3).

### 3.6. Differences in Mean PCT Levels between Uncomplicated Malaria, Asymptomatic Malaria, and Healthy Controls

The differences in PCT levels between uncomplicated malaria, asymptomatic malaria, and healthy controls were demonstrated in the study by Braun et al. [28]. They showed that the mean PCT levels were higher in asymptomatic malaria (25.27 ng/mL) than in asymptomatic malaria (0.42 ng/mL). They showed that the mean PCT levels were higher in uncomplicated malaria (25.27 ng/mL) compared to healthy control participants (0.64 ng/mL). Meanwhile, the mean PCT levels were lower in asymptomatic malaria (0.42 ng/mL) than in healthy control participants (0.64 ng/mL).

### 3.7. Other Information on PCT Levels in Patients with Malaria

Mbengue et al. [40] demonstrated significantly higher mean PCT levels in patients who died than those who survived (mean 53.6 ng/mL vs. 27.3 ng/mL). In addition, the study by Lubell et al. [38] showed that PCT levels were significantly higher in malaria infections than in viral infections.

### 3.8. Sensitivity Analysis

The meta-analysis of the differences in PCT levels between severe and uncomplicated malaria was tested for statistical validity using the fixed-effects model. The results show that patients with severe malaria had a higher mean of PCT than those with uncomplicated malaria (*p* < 0.001, WMD: 5.79, 95% CI: 4.82–6.77, I^2^: 84.6%, four studies, Supplementary Figure S2). By exclusion of the study by Braun et al. [28], the result shows that patients with severe malaria had a higher mean PCT level than those with uncomplicated malaria (*p*: 0.004, WMD: 10.4 ng/mL, 95% CI: 3.24–17.51, I^2^: 82.6%, three studies, Supplementary Figure S3).

### 3.9. Publication Bias

**Figure 4:**
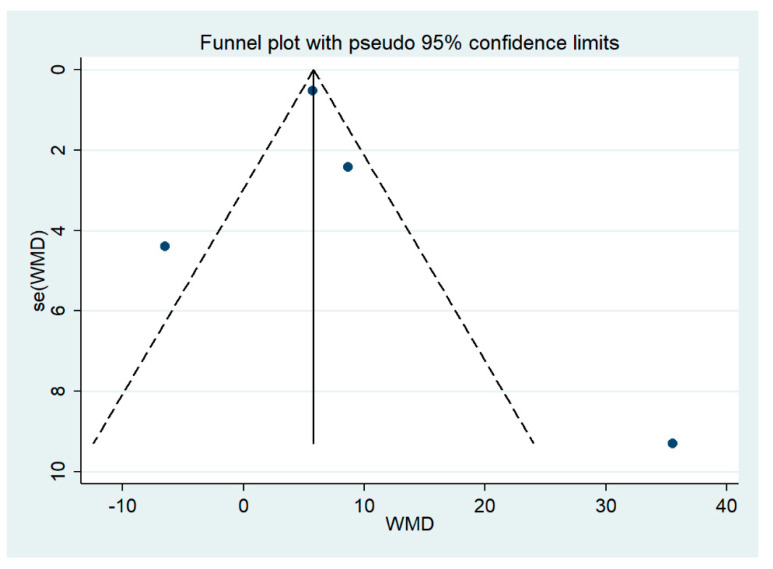
Funnel plot of included studies included in the meta-analysis between PCT levels (severe malaria and uncomplicated malaria). The funnel plots showed the asymmetrical distribution of WMD and the standard error (se) of WMD. Abbreviation: WMD, weighted mean difference; standard error (se).

The publication bias among the studies included for the meta-analysis of the difference in PCT levels between patients with severe malaria and uncomplicated malaria demonstrate the asymmetrical distribution of the effect estimates from the middle line (no effect size, Figure 4). Still, Egger’s test revealed no small study effect (*p*: 0.775) and indicated that publication bias among the included studies was less likely.

## 4. Discussion

A previous study found that patients with severe *P. falciparum* malaria had significantly higher PCT levels than those with uncomplicated *P. falciparum* malaria or non-*P. falciparum* malaria at admission [17]. The meta-analysis results reveal that the pooled mean PCT levels in patients with uncomplicated malaria were 3.92 ng/mL. Meanwhile, the pooled mean PCT levels in patients with severe malaria were 14.13 ng/mL. These results confirm that PCT levels were elevated in uncomplicated and severe malaria patients. In addition, patients with malaria had increased PCT levels which were caused by an increase in proinflammatory cytokine levels such as interleukin-6 (IL-6), tumor necrosis factor-α (TNF-α), and interleukin-1β (IL-β) during infections [42]. Serum PCT levels in healthy individuals are typically less than 0.1 ng/mL [43]. Therefore, increased PCT levels over the normal limit indicated mild infection; meanwhile, high PCT levels over 10 ng/mL might indicate severe infection [44]. The meta-analysis results of the difference in PCT levels between patients with severe and uncomplicated malaria revealed no difference in the mean PCT levels between the two groups. This result was in line with the most recent study showing that PCT levels were similar in both groups of participants [45].

The meta-analysis results could be explained by the number of studies included in the analysis being only four [16,28,29,30] which might cause a non-statistically significant difference in PCT levels. In addition, there were probable correlations between the levels of parasitemia, clinical symptoms, age, sex, immunity levels, and PCT levels among different clinical severities of malaria. For the level of parasitemia, the study by Braun et al. demonstrated that uncomplicated malaria was associated with high parasite counts as children with uncomplicated malaria had a higher parasite count (median 70,320 cell/μL) than those with severe malaria (median 17,150 cell/μL) [28]. This is the reason why the study by Braun et al. [28] showed a lower mean of PCT level in patients with severe malaria than those with uncomplicated malaria and resulted in an equal mean of PCT among those with severe malaria compared to those with uncomplicated malaria by the meta-analysis results. Meanwhile, other studies that reported higher PCT levels among patients with severe malaria than those with uncomplicated malaria demonstrated higher levels of parasitemia in patients with severe malaria than those with uncomplicated malaria [16,29,30]. For age groups, there was no difference in age and PCT levels among the outcomes of the meta-analysis as studies that enrolled both children and adults demonstrated higher PCT levels in severe cases than in uncomplicated cases [16,34,35]. In addition, there were comparable sex ratios among studies that showed higher PCT levels in severe cases than in uncomplicated cases [16,29,30]. For immunity levels across different groups of malaria, two studies with imported malaria with low immunity against malaria demonstrated higher PCT levels in severe cases than in uncomplicated cases [16,29,30]. Meanwhile, a study with endemic cases in Ghana where individuals had a high immunity against malaria, demonstrated lower PCT levels in severe cases than in uncomplicated cases [28]. Therefore, levels of parasitemia and immunity could explain the differences in PCT levels among severe and uncomplicated malaria in which low levels of parasitemia and immunity could contribute to the difference in PCT levels of patients with malaria. Furthermore, the sensitivity using the fixed-effect model and the meta-analysis of three studies by the one-leave-out method confirmed significantly higher mean PCT levels in patients with severe malaria than those with uncomplicated malaria.

A semi-quantitative PCT test is currently evaluated as a diagnostic tool for severe *P. falciparum* malaria. Using PCT as a point-of-care test may help clinicians efficiently identify patients with severe malaria. The previous study showed that PCT showed acceptable sensitivity and specificity in predicting severe falciparum malaria [29]. In addition, PCT was correlated with parasitemia and had better specificity at indicating severe falciparum malaria than CRP. The acceptable sensitivity required to predict severe falciparum malaria was a cut off at 5 ng/mL [29]. This cut-off showed a lower percentage of false positives and was suggested to be used in an area where malaria prevalence is low [29]. However, a higher cut-off value, such as at 2 ng/mL, was reported to have lower sensitivity (52%) with a specificity of 86% [46]. One study suggested that a cut-off value of 10 ng/mL more correctly identified patients with severe malaria who required critical care [41]. Another study indicated that a PCT cut-off point of 10 ng/mL was clinically valuable for identifying patients with severe *P. falciparum* malaria in low malaria settings [39].

PCT levels that correlated better with disease severity than CRP were reported in another cohort study that enrolled imported malaria [30]. Nevertheless, CRP was better than PCT at discriminating viral infections from bacterial infections [17]. PCT was more beneficial for critical care in the hospital context as it reacted faster to infections [10,35,36]. As PCT is also more rapidly eliminated, sharply decreasing PCT after treatment might be a marker for judging the curative effect of malaria as suggested by previous studies [36,39,40]. One study indicated that using PCT with a cut-off at 0.9 ng/mL in combination with CRP increased the correctness of identifying patients with severe malaria on admission than using CRP with other laboratory parameters such as copeptin, sodium, or sodium lactate [16]. From the results of the previous meta-analysis, PCT could guide the treatment of respiratory infections by reducing antibiotics [47,48]. Moreover, the previous study showed that PCT concentration at day 0 before treatment was a better predictive marker for fatality among patients with severe malaria [40]. One study found that PCT levels of more than 25 ng/mL were related to deaths [33]. Therefore, using PCT as a point-of-care test to identify patients with severe malaria benefits clinicians in decision-making and patients for receiving close monitoring.

This systematic review has several limitations. First, PCT was widely used in high-income countries. Therefore, the number of studies that reported the usefulness of PCT in malaria was limited, meaning that the result of the meta-analysis was based on a limited number of studies. Second, PCT levels in patients with malaria could be confounded by a concurrent bacterial infection (increased PCT levels) [49]. In addition, PCT levels between 0.5 and 2 ng/mL may be caused by other diseases or conditions such as antibiotics [50], malnourishment [51], acute respiratory illnesses [52], bacterial pneumonia and sepsis [53,54,55], and acute undifferentiated fever [56,57]. Other conditions such as acute myocardial infarction [58], trauma and post-operative surgery [59], or cardiogenic shock [60] may interfere with interpreting the results of PCT in malaria. Third, there was heterogeneity between the studies in the meta-analysis. However, the lack of publication bias among the studies included in the meta-analysis indicates that the analysis was robust. Finally, the difference in PCT levels among patients with uncomplicated and asymptomatic malaria could not be performed due to only one study by Braun et al., 2003 [28] that reported PCT levels in both groups.

## 5. Conclusions

There were probable correlations between the level of parasitemia, immunity level, and possibly bacterial or other parasitic co-infection that could affect PCT levels among different clinical severities of malaria. Therefore, the PCT level alone does not seem to be a suitable biomarker to discriminate between the severe/uncomplicated and infected/uninfected cases. Therefore, further studies should investigate the increased PCT levels in combination with other markers associated with malaria infection and severity.

## Figures and Tables

**Table 1:** Characteristics of the included studies.

No.	Reference Study	Study Design	Study Location	Year	Characteristics of Participants	Number of Participants	*Plasmodium* Spp.	Mean Age (Years or Months)	Age Groups	Male Percentage	Procalcitonin (Mean ± SD or Median, Range) (ng/mL)	Parasite Density (Per Microliter)	Method for Malaria Detections	Method for Procalcitonin
1	Braun et al., 2003 [28]	Case-control study	Ghana	NS	Severe malaria (18), Uncomplicated malaria (18), Asymptomatic malaria (21), Healthy controls (13)	70	*P. falciparum*	Severe malaria (18): mean 20.9 months (95%CI 6.6–35.2), Uncomplicated malaria (18): 44.2 (32.2–56.3), Asymptomatic malaria (21): 67.3 (55.5–79.1), Healthy controls (13): 77.5 (58.2–96.9)	Children	NS	Severe malaria (18): 18.77 ± 11.60, Uncomplicated malaria (18): 25.27 ± 14.55, Asymptomatic malaria (21): 0.42 ± 0.27, Healthy controls (13): 0.64 ± 0.32	Severe malaria (18): mean 17,150 (5394–28,910), Uncomplicated malaria (18): 70,320 (36,380–104,300), Asymptomatic malaria (21): 1709 (1136–2282)	Microscopy	LUMI-Test (Brahms Hennigsdorf, Germany)
2	Bruneel et al., 2016 [30]	Cohort study	France	2007–2010	Severe malaria (155), Uncomplicated malaria (140)	295	*P. falciparum*	Severe malaria (155): 44.4 ± 14.0, Uncomplicated malaria (140): 39.3 ± 12.5	Adults	Severe malaria (155): 67.1, Uncomplicated malaria (140): 71.4	Severe malaria (155): 41.1 ± 115.2, Uncomplicated malaria (140): 5.6 ± 11.2	Severe malaria (155): 8.0% (3.3–15.0), Uncomplicated malaria (140): 0.5% (0.1–2.3)	Microscopy	PCT sensitive Kryptor Analyzer (Brahms, Hennigsdorf, Germany)
3	Chiwakata et al., 2001 [33]	Prospective observational study	Germany	NS	Severe malaria (25), Uncomplicated malaria (36)	61	*P. falciparum*	NS	NS	NS	Severe malaria (25): 10.67 (2.61–132.1), Uncomplicated malaria (36): 1.52 (0.5–5.6)	NS	Microscopy	Immunoluminometric assay (LUMItest PCT, (BRAHMS Diagnostica))
4	Erdman et al., 2011 [37]	Case-control study	Uganda	2007–2009	Uncomplicated malaria (53), Cerebral malaria (44), Severe malarial anemia (59)	156	*P. falciparum*	Uncomplicated malaria (53): 4.4 (2.1–8.1), Cerebral malaria (44): 3.0 (1.5–4.3), Severe malarial anemia (59): 1.3 (0.9–2.0)	6 months and 12 years	Uncomplicated malaria (53): 54.7, Cerebral malaria (44): 47.7, Severe malarial anemia (59): 50.8	PCT was elevated in children with severe malaria compared to uncomplicated malaria	Uncomplicated malaria (53): 38,600 (16,600–126,000), Cerebral malaria (44): 98,600 (15,600–276,000), Severe malarial anemia (59): 266,00 (7460–126,000)	Microscopy	ELISAs (Ray BioTech)
5	Hesselink et al., 2009 [39]	Prospective observational study	Netherlands	NS	Severe falciparum malaria (6), Uncomplicated falciparum malaria (65), Uncomplicated non-falciparum malaria (29)	100	*P. falciparum* and non-*P. falciparum* (*P. vivax, P. ovale, P. malariae*)	Severe falciparum malaria (6): 48 (29–55), Uncomplicated falciparum malaria (65): 36 (9–67), Uncomplicated non-falciparum malaria (29): 36 (15–62)	Adults	Severe falciparum malaria (6): 66.7, Uncomplicated falciparum malaria (65): 72.3, Uncomplicated non-falciparum malaria (29): 75.9	Severe falciparum malaria (6): increased 6/6, Uncomplicated falciparum malaria (65): 42/65, Uncomplicated non-falciparum malaria (29): 25/29	Severe falciparum malaria (6): 174,900 (80,500–567,000), Uncomplicated falciparum malaria (65): 3422 (23–385,000), Uncomplicated non-falciparum malaria (29): NS	Microscopy, RDT, QBC (Quantitative Buffy Coat)	BRAHMS PCT-Q^®^ test (Brahms Diagnostics, Germany)
6	Hollenstein et al., 1998 [36]	Case-control study	Thailand	NS	Severe malaria (27)	27	*P. falciparum*	24.8 ± 8.3	16–45	85.2	40 ng/mL (range 0.04–662), elevated 26/27	290,680 (533–1,147,040)	Microscopy	Immunoluminometric assay (LUMItest, PCT; Brahms Diagnostica GmbH, Berlin, Germany)
7	Huang et al., 2019 [34]	Retrospective study	Congo, Angola, Pakistan, African countries	2014–2018	Severe malaria (1), Uncomplicated malaria (21)	22	*P. falciparum* (19), *P. vivax* (2), *P. ovale* (1)	27.2 ± 4.8	20–48	100	3.28 (0.95, 13.11), elevated 21/22	NS	Microscopy and RDT	A Roche Cobas E601 automatic electrochemical luminescence analyzer and an auxiliary reagent (ElecsysBRAHMSprocalcitonin, RocheDiagnostics GmbH)
8	Lin et al., 2018 [31]	Retrospective study	China	2007–2016	Severe malaria (27): exchange transfusion (15), controls (12)	27	*P. falciparum*	Exchange transfusion (15): 47.8 ± 4.4, controls (12): 46.0 ± 5.8	NS	NS	Exchange transfusion (15): 53.83 ± 29.41	Exchange transfusion (15): 742,000 ± 518,000, controls (12): 587,000 ± 264,000	NS	NS
9	Lubell et al., 2015 [38]	Retrospective study	Cambodia, Laos and, Thailand	NS	Uncomplicated malaria (125)	125	NS	NS	NS	NS	Procalcitonin levels were significantly higher in malaria infections as compared with viral infections	NS	NS	Enzyme-Linked Fluorescent Assay technique via the Mini-VIDAS platform (BioMérieux, 69,280 Marcy-l’Etoile, France)
10	Mbengue et al., 2011 [40]	Prospective observational study	Senegal	2000–2003	Cerebral malaria (98)	98	*P. falciparum*	NS	NS	NS	Mean PCT levels were elevated in patients with active infection, with a large range of values (0.1 to 280 nanog per mL) significantly higher on day 0 in fatal cases than in survival cases (53.6 vs. 27.3; *p* = 0.01).	NS	Microscopy and RDT	Double site sandwich immunoluminometric assay according to the manufacturer’s instructions (LUMI-test^®^ PCT; Brahms Diagnostica Gmbh, Berlin, Germany)
11	Mohapatra et al., 2013 [41]	Prospective observational study	India	2011–2012	Severe malaria (41), Uncomplicated malaria (19)	60	*P. falciparum*	Severe malaria (41): 37.10 ± 13.238, Uncomplicated malaria (19): 37.84 ± 15.5	Adults	Severe malaria (41): 56.1, Uncomplicated malaria (19): 63.2	Severe malaria (41): increased 41/41, Uncomplicated malaria (19): 12/19	Severe malaria (41): 8578.82 ± 214.56, Uncomplicated malaria (19): 4569.58 ± 178.9	Microscopy	B·R·A·H·M·S PCT-Q (B·R·A·H·M·S, Aktiengesellschaft Neuendorfstrasse 25 D-16761 Hennigsdorf, Germany)
12	Prodjosoewojo et al., 2019 [32]	Cohort study	Indonesia	2004–2016	Patients with malaria (4)	4	NS	25 (19.8–35)	NS	75	34.2 (20.7–43)	NS	Microscopy	BRAHMS PCT, Elecsys and Cobas e 411 analyzers (Roche Diagnostics GmbH, US)
13	Righi et al., 2016 [29]	Prospective observational study	Italy	2011–2013	Severe malaria (9), Uncomplicated malaria (21)	30	*P. falciparum*	Severe malaria (9): 42 ± 13.2, Uncomplicated malaria (21): 41 ± 9.9	Adults	Severe malaria (9): 89, Uncomplicated malaria (21): 81	Severe malaria (9): 6.88 (4–9), Uncomplicated malaria (21): 0.38 (0.30–2.72)	Severe malaria (9): 6.25% (3.25–13), Uncomplicated malaria (21): 1% (0.3–1.5)	Microscopy	Automated immunofluorescent assays (B·R·A·H·M·S PCT sensitive KRYPTOR, Brahms Diagnostics, Germany)
14	Uzzan et al., 2006 [35]	Prospective observational study	France	NS	Severe malaria (3), Uncomplicated malaria (25)	28	*P. falciparum*	37 ± 13	Adults	64.3	Malaria (18): 3.0 ± 4.6, increased in 14/18	NS	Microscopy	Immunoluminometric assay on antibody-coated tubes (Lumitestw PCT, BRAHMS Diagnostica GmbH, Berlin, Germany)
15	van Wolfswinkel et al., 2012 [16]	Cohort study	Netherlands	1999–2010	Severe falciparum malaria (25), Uncomplicated falciparum malaria (116), Uncomplicated non-falciparum malaria (63)	204	*P. falciparum* and non-*P. falciparum* (*P. vivax, P. ovale, P. malariae*)	Severe falciparum malaria (25): 44 (23–70), Uncomplicated falciparum malaria (116): 41 (11–69), Uncomplicated non-falciparum malaria (63): 38 (8–62)	Adults	Severe falciparum malaria (25): 60, Uncomplicated falciparum malaria (116): 79, Uncomplicated non-falciparum malaria (63): 70	Severe falciparum malaria (25): 1.9 (0.9–42.3), Uncomplicated falciparum malaria (116): 0.6 (0.0–11.2), Uncomplicated non-falciparum malaria (63): 1.6 (0.0–42.6)	Severe falciparum malaria (25): 284,005 (39,600–1,380,600), Uncomplicated falciparum malaria (116): 22,657 (2–156,600), Uncomplicated non-falciparum malaria (63): NS	Microscopy, RDT, QBC (Quantitative Buffy Coat)	EIA test (VIDAS BRAHMS Procalcitonin, bioMérieux, Lyon, France)

Abbreviations: NS, not specified; RDT, rapid diagnostic test; QBC, quantitative buffy coat.

## Data Availability

All data related to the manuscript are available in the main manuscript and its supplementary files.

## Supplementary Materials

The following supporting information can be downloaded at: https://www.mdpi.com/article/10.3390/ijerph191811389/s1, Supplementary Figure S1: Forrest plot demonstrated the pooled mean PCT levels in patients with uncomplicated malaria; Supplementary Figure S2: Forrest plot demonstrated the difference in the mean PCT levels (ng/mL) between patients with severe malaria and uncomplicated malaria by the fixed-effect model; Supplementary Figure S3: Forrest plot demonstrated the difference in the mean PCT levels (ng/mL) between patients with severe malaria and uncomplicated malaria (sensitivity analysis); Table S1: Search terms; Table S2: Quality of the included studies; PRISMA 2020 Abstract Checklist; PRISMA 2020 Checklist.
